# Effects of COVID-19 on career and specialty choices among Chinese medical students

**DOI:** 10.1080/10872981.2021.1913785

**Published:** 2021-04-14

**Authors:** Jiahui Deng, Jianyu Que, Suying Wu, Yingjian Zhang, Jiajia Liu, Sijing Chen, Yongxi Wu, Yimiao Gong, Siwei Sun, Kai Yuan, Yanping Bao, Maosheng Ran, Jie Shi, Yun Kwok Wing, Le Shi, Lin Lu

**Affiliations:** a Peking University Sixth Hospital, Peking University Institute of Mental Health, NHC Key Laboratory of Mental Health (Peking University), National Clinical Research Center for Mental Disorders (Peking University Sixth Hospital), Peking University, Beijing, China; b Xiamen Xianyue Hospital, Xiamen, China; c National Institute on Drug Dependence and Beijing Key Laboratory on Drug Dependence Research, Peking University, Beijing, China; d Department of Psychiatry, The Chinese University of Hong Kong, Shatin, Hong Kong; e Fujian Provincial Hospital, Fuzhou, China; f Peking-Tsinghua Center for Life Sciences and PKU-IDG/McGovern Institute for Brain Research, Beijing, China; g Department of Social Work and Social Administration, University of Hong Kong, Hong Kong, China

**Keywords:** COVID-19, career choice, specialty choice, medical students, psychological problems

## Abstract

Coronavirus disease 2019 (COVID-19) pandemic has brought unprecedented challenges to medical education systems and medical students. The aim of this study was to investigate the effects of COVID-19 pandemic on medical career and specialty choices among medical students. An online cross-sectional survey of Chinese medical students was conducted during the COVID-19 pandemic from February to April 2020. The students’ willingness to be a doctor before and after the COVID-19 pandemic and changed willingness to specialize in respiratory medicine and infectious diseases were investigated. Multiple linear regression and binary logistic regression was used to explore factors that were associated with changes of willingness. A total of 1,837 medical students, including 1,227 females (66.8%), with a median age of 21.0 years, were recruited. Of the participants, 10.6% and 6.9% showed increased and decreased willingness to be a doctor after the COVID-19 outbreak, respectively. Moreover, 11.7% showed increased willingness and 9.5% showed decreased willingness to major in respiratory medicine and infectious diseases. Students with younger age, lower household income, fewer depressive symptoms, less exposure to negative pandemic information and more satisfaction with their own major after the pandemic were associated with increased willingness to be a doctor. Students who engaged in regular exercise, were males and undergraduate level, were interested in medicine, paid more attention to positive information, were satisfied with their majors, and had increased willingness to be a doctor after the pandemic were more likely to choose to specialize in respiratory medicine and infectious disease. However, the severity of anxiety symptoms was associated with decreased willingness to work in the specialties of respiratory medicine and infectious diseases. Psychological problems and professional satisfaction appear to be independent factors that affect medial career and specialty choices. The impacts of the COVID-19 pandemic on medical students require further research.

## Introduction

Coronavirus disease 2019 (COVID-19) has been continuously spreading since the World Health Organization declared it a ‘public health emergency of international concern’ in late January 2020[1]. As of 13 November 2020, there have been more than 52.75 million cases of infection in 191 countries and territories worldwide[2]. COVID-19 pandemic has brought unprecedented challenges to the medical education system and medical students.

Most countries implemented social distancing policies, such as school and university closures, to control transmission during the pandemic. The COVID-19 pandemic and subsequent cancellation of clinical clerkships and rotations has posed unique challenges to students’ education, which may affect their occupational belief formation. Although medical students may have more time to consider their own interests in terms of subspecialty selection, their opportunities to gain a deep understanding of the specialty of respiratory medicine and infectious diseases though clinical clerkship and rotation decreased during the pandemic[3]. In addition to the adverse impacts, there were also positive impacts of the pandemic on medical students in terms of the opportunities to solve the puzzles and mysteries of emerging infections and the encouragement to pursue altruistic and dedicated healthcare careers in a more determined way[4].

Negative life events, such as the experience of the COVID-19 pandemic, have been demonstrated to have a strong relationship with the mental health status of medical students[5]. The pandemic and changes in educational patterns also had psychological effects on medical students[6]. Approximately 30% of medical students experienced psychological distress during the pandemic, and this distress was associated with several specific COVID-related stressors [7,8]. More notably, 68% of medical students reported deterioration in their mental health since the onset of the COVID-19 pandemic[9]. Both the COVID-19 pandemic and its related mental repercussions may exert some influence on students’ future medical career choices. Psychological problems are related to decreased interest and increased regret of their career choice, and dropout rates among medical students have increased [10,11]. Twenty percent of medical students believe that the COVID-19 pandemic will affect their choice of specialty[12]. Approximately one-third of medical students preferred not to return to the clinical setting after the pandemic[13]. Furthermore, witnessing the stress of frontline workers and the psychological distress from which they suffer while fighting the COVID-19 pandemic may also raise concern about the potential occupational hazards of medical professionals. Over one-third of health care workers (HCWs) suffer from psychological distress when exposed to a high risk of infection, heavy work intensity and insufficient medical supplies during the COVID-19 pandemic [14–16]. Medical students, who are the reserve force for developing medical and health services in China, may experience anticipated worries and increased stress after witnessing HCWs who are exposed to substantial stress.

By the end of 2019, the number of licensed doctors in China was approximately 3.87 million[17]. For every 1000 people, there were 2.77 doctors (including assistant practitioners) in China, and this number was lower than the 3.5 doctors per 1000 people in Organization for Economic Co-operation and Development Countries[18]. One preliminary study found that only 752,233 (15.91%) of 4,727,977 Chinese clinical medical graduates registered in clinical practice became practicing doctors during the period from 2005 to 2014[19]. In addition, only one-sixth of the 600,000 newly qualified young doctors have been registered at a healthcare institution during a 5-year period[20]. Possible reasons for this extremely low rate are that many Chinese medical graduates want to teach or conduct research at medical universities with a lower work intensity or to work at pharmaceutical companies with a higher salary. More notably, only 7% of internal medicine residents applied to the specialty of infectious diseases, and most of them decided to work in this field at the early stage of their medical education[21].

However, very limited studies have investigated differences in career choice among Chinese medical students before and after the COVID-19 outbreak. One previous study found that COVID-19 might have a positive impact on career choice by strengthening pediatric medical students’ beliefs and choices to become good doctors[4]. The impact of COVID-19 on the career choices of medical students, as well as the related factors, remain unclear. Thus, we conducted a cross-sectional survey to explore the changes and factors that influence medical students’ careers and specialty choices before and after the COVID-19 pandemic.

## Materials and methods

### Study design

During the COVID-19 pandemic in China, an online cross-sectional study with convenience sampling was conducted from February 2020 to April 2020 through a platform called Questionnaire Star (www.wjx.cn). Medical students across the country were invited to participate in this survey via social networks using the snowball technique, and participants received screening results when they completed this survey.

All procedures were in accordance with the ethical standards of the Peking University Sixth Hospital Institutional Review Board (No. 2020-2-21-1) and with the Helsinki Declaration of 1975, as revised in 2000. Oral informed consent was obtained from each participant being included in this study.

#### Study population

Medical students over 18 years old from China were invited to participate in this survey. A total of 4,543 individuals clicked on the survey link, and 1,844 medical students completed the survey, with a response rate of 40.6%. Seven students under the age of 18 years old were excluded, and 1,837 medical students from 31-province level regions were included in the final analysis. To produce a more representative sample, we monitored of the distribution of questionnaires received over time. If the received questionnaires in specific regions were relatively few, we would send the designated link to social networks of investigators and research teams who can help us invite more participants to complete this survey there. The proportions of participants distributed in the east, west, central and northeast areas were 32.3%, 41.0%, 16.5% and 7.8%, respectively. A total of 2.4% individuals did not report their regions. The low proportion in the northeastern area may be due to the limited number of medical universities.

#### Measurements and covariates

Demographic variables, i.e., age, gender, ethnicity, residence, educational level, annual family income and regular exercise habits, were collected. Changes in willingness to be a doctor before and after the COVID-19 pandemic were assessed by the 0- to 10-point visual analogue scale (VAS), which required participants to subjectively report the extent of their willingness to be a doctor before and after the COVID-19 pandemic. Participants were categorized into three groups as follow: decreased group, VAS score before pandemic was higher than after pandemic; no change group, VAS score before pandemic was equal to after pandemic; increased group, VAS score before pandemic was lower than after pandemic. In addition, changes in willingness to specialize in respiratory medicine and infectious diseases were investigated by an item with 3 levels: 1, decreased willingness; 2, no change; 3, increased willingness.

Exposure to pandemic-related factors was investigated by asking the following questions: ‘What type of pandemic information do you pay more attention to?’ and ‘Did you receive negative feedback from your families and friends who were working on the frontline?’. Questions were also asked about the impacts of having relatives and friends with suspected or confirmed COVID-19 infection. Additionally, reasons for majoring in medicine were investigated by true or false questions: ‘Did you follow your own inclinations when choosing to major in medicine?’, ‘Are you interested in medicine?’, and ‘Were you influenced by your elders (parents or other relatives) when selecting medicine as your major?’.

Depression, anxiety and insomnia symptoms were assessed by the Chinese version of the Patient Health Questionnaire (PHQ-9), the Generalized Anxiety Disorder Scale (GAD-7) and the Insomnia Severity Index (ISI), respectively. Cutoff points of 5, 10 and 15 on the PHQ-9 and GAD-7 scores were defined as mild, moderate and severe symptoms of depression and anxiety [22,23]. Scores of 8, 15 and 22 on the ISI were used as cutoff points to classify insomnia severity into mild, moderate and severe levels [15,16].

#### Data analysis

Non-normally distributed continuous variables are presented as the medians with interquartile ranges (IQRs), and categorical variables are presented as numbers with percentages. Mann-Whitney tests and χ^2^ tests were used to assess the significant difference of non-normally distributed continuous variables and categorical variables. Factors that were associated with changed willingness to be a doctor were explored by multiple linear regression analysis. Factors that were associated with decreased or increased willingness were explored by separated binary logistic regression after testing the proportional odds assumption is not fulfilled in ordinal logistic regression model[24]. Unadjusted and adjusted coefficients with 95% confidence intervals (CIs) were calculated by univariate and multivariate linear regression analysis, unadjusted and adjusted ORs with 95% CIs were calculated by univariate and multivariate logistic regression analysis. All the data analyses were conducted using SPSS 20.0 software. All the tests were two-sided, with a p value less than 0.05 considered significant.

## Results

### Descriptive characteristics

The demographic characteristics of the participants and the distribution of changes in willingness to be a doctor and to specialize in respiratory medicine and infectious diseases after the COVID-19 pandemic are shown in (Tables 1 and 2). The survey included 1,837 medical students (1,227 [66.8%] women and 610 [33.2%] men) aged 18 and over. The median (IQR) age was 21.0 (20.0–25.0) years. Of the participants, 82.1% were from the Han population, 82.8% were undergraduate students, 37.0% had a habit of regular exercising, and 75.4% had an annual household income of no more than 100 thousand yuan. Only a small portion of students were exposed to adverse events related to the COVID-19 pandemic, i.e., living in the most affected pandemic area (2.0%), paying more attention to negative pandemic information (4.4%), obtaining negative feedback from families and friends who joined frontline work (0.9%), and having relatives who had suspected or confirmed COVID-19 infection (3.4%). A total of 43.1% of students reported depressive symptoms, 40.8% reported anxiety symptoms, and 25.6% reported insomnia symptoms. Overall, 10.6% of the medical students showed increased willingness to be a doctor after the COVID-19 pandemic, while 6.9% had decreased willingness. Additionally, 11.7% showed increased willingness to select respiratory medicine and infectious diseases as their majors, while 9.5% showed decreased willingness. Most of the students who selected medicine as their career followed their own wishes (83.7%) and were interested in medicine (91.1%). Nearly half of the students made their medical career decisions under pressure from their elders, and only 14.3% were more satisfied with their majors after the COVID-19 pandemic.

**Table 1.** Characteristics of participants according to indicators of changes in willingness to be a doctor after the COVID-19 pandemic

Variables	Total (n = 1,837; 100.0%)	Changes in willingness to be a doctor			
		Increased willingness (n = 194; 10.6%)	No change (n = 1,517; 82.5%)	Decreased willingness (n = 126; 6.9%)	*P*
Median (IQR) age	21.0(20.0–25.0)	22.0(20.0–24.0)	21.0(20.0–25.0)	23.5(20.8–27.0)	<0.001
The median (IQR) willingness to be a doctor before the COVID-19 pandemic	9.0(7.0–10.0)	7.0(5.0–8.0)	9.0(8.0–10.0)	8.0(7.0–9.0)	<0.001
Sex					0.753
Men	610(33.2%)	65(33.5%)	507(33.4%)	38(30.2%)	
Women	1,227(66.8%)	129(66.5%)	1,010(66.6%)	88(69.8%)	
Ethnicity					0.351
Han Chinese	1,508(82.1%)	154(79.4%)	1,246(82.1%)	108(85.7%)	
Other	329(17.9%)	40(20.6%)	271(17.9%)	18(14.3%)	
Education level					0.270
Undergraduate	1,521(82.8%)	159(82.0%)	1,264(83.3%)	98(77.8%)	
Graduate	316(17.2%)	35(18.0%)	253(16.7%)	28(22.2%)	
Annual household income (¥)					0.014
≤100 thousand	1,386(75.4%)	159(82.0%)	1,143(75.3%)	84(66.7%)	
100–300 thousand	356(19.4%)	32(16.5%)	292(19.2%)	32(25.4%)	
>300 thousand	95(5.2%)	3(1.5%)	82(5.4%)	10(7.9%)	
Habit of regular exercising					0.935
No	1,158(63.0%)	124(63.9%)	956(63.0%)	78(61.9%)	
Yes	679(37.0%)	70(36.1%)	561(37.0%)	48(38.1%)	
Exposure to the most affected pandemic area					0.030
No	1,800(98.0%)	188(96.9%)	1,492(98.4%)	120(95.2%)	
Yes	37(2.0%)	6(3.1%)	25(1.6%)	6(4.8%)	
Preferential attention toward pandemic information					0.002
Negative	81(4.4%)	13(6.7%)	58(3.8%)	10(7.9%)	
Neutral	690(37.6%)	79(40.7%)	551(36.3%)	60(47.6%)	
Positive	1,066(58.0%)	102(52.6%)	908(59.9%)	56(44.4%)	
Did you receive negative feedback from your families and friends who joined the frontline work?					0.015
No	1,821(99.1%)	193(99.5%)	1,506(99.3%)	122(96.8%)	
Yes	16(0.9%)	1(0.5%)	11(0.7%)	4(3.2%)	
Was anyone close to you suspected or confirmed to be infected?					0.680
No	1,774(96.6%)	187(96.4%)	1,467(96.7%)	120(95.2%)	
Yes	63(3.4%)	7(3.6%)	50(3.3%)	6(4.8%)	
Did you follow your own inclinations when choosing to major in medicine?					<0.001
No	299(16.3%)	54(27.8%)	210(13.8%)	35(27.8%)	
Yes	1,538(83.7%)	140(72.2%)	1,307(86.2%)	91(72.2%)	
Are you interested in medicine?					<0.001
No	163(8.9%)	36(18.6%)	113(7.4%)	14(11.1%)	
Yes	1,674(91.1%)	158(81.4%)	1,404(92.6%)	112(88.9%)	
Were you influenced by your elders (parents or other relatives) when selecting medicine as your major?					0.112
No	1,034(56.3%)	122(62.9%)	846(55.8%)	66(52.4%)	
Yes	803(43.7%)	72(37.1%)	671(44.2%)	60(47.6%)	
Changes in professional satisfaction after the COVID-19 pandemic					<0.001
Less satisfied	128(7.0%)	22(11.3%)	74(4.9%)	32(25.4%)	
No change	1,446(78.7%)	103(53.1%)	1,265(83.4%)	78(61.9%)	
More satisfied	263(14.3%)	69(35.6%)	178(11.7%)	16(12.7%)	
Scores of PHQ-9	3.0(0.0–8.0)	4.0(1.0–8.0)	3.0(0.0–8.0)	6.5(1.0–9.0)	<0.001
Symptoms of depression					<0.001
No	1,046(56.9%)	100(51.5%)	895(59.0%)	51(40.5%)	
Mild	579(31.5%)	67(34.5%)	466(30.7%)	46(36.5%)	
Moderate	124(6.8%)	15(7.7%)	95(6.3%)	14(11.1%)	
Severe	88(4.8%)	12(6.2%)	61(4.0%)	15(11.9%)	
Scores of GAD-7	3.0(0.0–7.0)	5.0(1.0–7.0)	3.0(0.0–7.0)	5.5(1.8–8.0)	<0.001
Symptoms of anxiety					<0.001
No	1,088(59.2%)	94(48.5%)	939(61.9%)	55(43.7%)	
Mild	582(31.7%)	81(41.8%)	455(30.0%)	46(36.5%)	
Moderate	132(7.2%)	15(7.7%)	98(6.5%)	19(15.1%)	
Severe	35(1.9%)	4(2.1%)	25(1.6%)	6(4.8%)	
Scores of ISI	4.0(1.0–8.0)	5.0(1.0–9.0)	4.0(1.0–7.0)	6.0(2.0–10.0)	<0.001
Symptoms of insomnia					0.018
No	1,367(74.4%)	137(70.6%)	1,152(75.9%)	78(61.9%)	
Subclinical	366(19.9%)	43(22.2%)	287(18.9%)	36(28.6%)	
Moderate	89(4.8%)	13(6.7%)	66(4.4%)	10(7.9%)	
Severe	15(0.8%)	1(0.5%)	12(0.8%)	2(1.6%)	

GAD-7, Generalized Anxiety Disorder Scale; IQR, Interquartile range; ISI, Insomnia Severity Index; PHQ-9, Patient Health Questionnaire-9 item

**Table 2.** Characteristic of participants according to indicators of changes in willingness to specialize in respiratory medicine and infectious diseases after the COVID-19 pandemic

Variables	Total (n = 1,837; 100.0%)	Changes in willingness to specialize in respiratory medicine and infectious diseases			
		Increased willingness (n = 215; 11.7%)	No change (n = 1,448; 78.8%)	Decreased willingness (n = 174; 9.5%)	*P*
Median (IQR) age	21.0(20.0–25.0)	21.0(19.0–23.0)	21.0(20.0–25.0)	23.0(21.0–26.0)	<0.001
Sex					0.005
Men	610(33.2%)	92(42.8%)	459(31.7%)	59(33.9%)	
Women	1,227(66.8%)	123(57.2%)	989(68.3%)	115(66.1%)	
Ethnicity					0.010
Han Chinese	1,508(82.1%)	166(77.2%)	1,187(82.0%)	155(89.1%)	
Other	329(17.9%)	49(22.8%)	261(18.0%)	19(10.9%)	
Education level					<0.001
Undergraduate	1,521(82.8%)	198(92.1%)	1,197(82.7%)	126(72.4%)	
Graduate	316(17.2%)	17(7.9%)	251(17.3%)	48(27.6%)	
Annual household income (¥)					0.002
≤100 thousand	1,386(75.4%)	177(82.3%)	1,093(75.5%)	116(66.7%)	
100–300 thousand	356(19.4%)	29(13.5%)	286(19.8%)	41(23.6%)	
>300 thousand	95(5.2%)	9(4.2%)	69(4.8%)	17(9.8%)	
Habit of regular exercising					<0.001
No	1,158(63.0%)	107(49.8%)	930(64.2%)	121(69.5%)	
Yes	679(37.0%)	108(50.2%)	518(35.8%)	53(30.5%)	
Exposure to the most affected pandemic area					0.586
No	1,800(98.0%)	212(98.6%)	1,419(98.0%)	169(97.1%)	
Yes	37(2.0%)	3(1.4%)	29(2.0%)	5(2.9%)	
Preferential attention toward pandemic information					<0.001
Negative	81(4.4%)	2(0.9%)	60(4.1%)	19(10.9%)	
Neutral	690(37.6%)	56(26.0%)	552(38.1%)	82(47.1%)	
Positive	1,066(58.0%)	157(73.0%)	836(57.7%)	73(42.0%)	
Did you receive negative feedback from your families and friends who joined the frontline work?					<0.001
No	1,821(99.1%)	215(100.0%)	1,438(99.3%)	168(96.6%)	
Yes	16(0.9%)	0(0.0%)	10(0.7%)	6(3.4%)	
Was anyone close to you suspected or confirmed to be infected?					<0.001
No	1,774(96.6%)	208(96.7%)	1,409(97.3%)	157(90.2%)	
Yes	63(3.4%)	7(3.3%)	39(2.7%)	17(9.8%)	
Did you follow your own inclinations when choosing to major in medicine?					<0.001
No	299(16.3%)	18(8.4%)	230(15.9%)	51(29.3%)	
Yes	1,538(83.7%)	197(91.6%)	1,218(84.1%)	123(70.7%)	
Are you interested in medicine?					<0.001
No	163(8.9%)	4(1.9%)	132(9.1%)	27(15.5%)	
Yes	1,674(91.1%)	211(98.1%)	1,316(90.9%)	147(84.5%)	
Were you influenced by your elders (parents or other relatives) when selecting medicine as your major?					0.002
No	1,034(56.3%)	100(46.5%)	845(58.4%)	89(51.1%)	
Yes	803(43.7%)	115(53.5%)	603(41.6%)	85(48.9%)	
Changes in willingness to be a doctor after the COVID-19 pandemic					<0.001
Decreased willingness	126(6.9%)	13(6.0%)	71(4.9%)	42(24.1%)	
No change	1,517(82.5%)	169(78.6%)	1,231(85.0%)	117(67.2%)	
Increased willingness	194(10.6%)	33(15.3%)	146(10.1%)	15(8.6%)	
Changes in professional satisfaction after the COVID-19 pandemic					<0.001
Less satisfied	128(7.0%)	17(7.9%)	82(5.7%)	29(16.7%)	
No change	1,446(78.7%)	151(70.2%)	1,177(81.3%)	118(67.8%)	
More satisfied	263(14.3%)	47(21.9%)	189(13.1%)	27(15.5%)	
Scores of PHQ-9	3.0(0.0–8.0)	2.0(0.0–9.0)	3.0(0.0–8.0)	7.0(2.0–9.0)	<0.001
Symptoms of depression					<0.001
No	1,046(56.9%)	127(59.1%)	856(59.1%)	63(36.2%)	
Mild	579(31.5%)	63(29.3%)	441(30.5%)	75(43.1%)	
Moderate	124(6.8%)	17(7.9%)	88(6.1%)	19(10.9%)	
Severe	88(4.8%)	8(3.7%)	63(4.4%)	17(9.8%)	
Scores of GAD-7	3.0(0.0–7.0)	2.0(0.0–7.0)	3.0(0.0–7.0)	7.0(3.0–9.0)	<0.001
Symptoms of anxiety					<0.001
No	1,088(59.2%)	133(61.9%)	898(62.0%)	57(32.8%)	
Mild	582(31.7%)	63(29.3%)	434(30.0%)	85(48.9%)	
Moderate	132(7.2%)	17(7.9%)	91(6.3%)	24(13.8%)	
Severe	35(1.9%)	2(0.9%)	25(1.7%)	8(4.6%)	
Scores of ISI	4.0(1.0–8.0)	3.0(1.0–7.0)	4.0(1.0–7.0)	6.5(3.0–10.0)	<0.001
Symptoms of insomnia					<0.001
No	1,367(74.4%)	164(76.3%)	1,091(75.3%)	112(64.4%)	
Subclinical	366(19.9%)	43(20.0%)	279(19.3%)	44(25.3%)	
Moderate	89(4.8%)	7(3.3%)	70(4.8%)	12(6.9%)	
Severe	15(0.8%)	1(0.5%)	8(0.6%)	6(3.4%)	

GAD-7, Generalized Anxiety Disorder Scale; IQR, Interquartile range; ISI, Insomnia Severity Index; PHQ-9, Patient Health Questionnaire-9 item

## Factors independently associated with changed willingness to be a doctor

Multivariate linear regression analysis showed that having a higher willingness to be a clinical doctor before the COVID-19 pandemic was associated with a lower risk of altered willingness to be a doctor after the COVID-19 pandemic (β = −0.107, 95%CI: −0.127 to −0.087, *p* < 0.001). Individuals who were more satisfied with their majors (β = 0.364, 95%CI: 0.253 to 0.475, *p* < 0.001) after the COVID-19 pandemic were positively associated with increased willingness to be a doctor, but with less satisfaction (β = −0.348, 95%CI: −0.501 to −0.195, *p* < 0.001) were negatively associated with increased willingness. Medical students with an annual household income over 300 thousand yuan had a lower rate of increased willingness of being a doctor after the COVID-19 pandemic than those with an annual income no more than 100 thousand yuan (β = −0.261, 95%CI: −0.437 to −0.085, *p* = 0.004), and the severity of depressive symptoms (β = −0.011, 95%CI: −0.019 to −0.003, *p* = 0.009) was negatively associated with increased willingness to be a doctor after the COVID-19 pandemic. Individuals who were older (β = −0.015, 95%CI: −0.025 to −0.004, *p* = 0.007) and female (β = −0.099, 95%CI: −0.181 to −0.018, *p* = 0.017) were less likely to increase willingness after pandemic. In addition, receiving negative feedback (β = −0.595, 95%CI: −1.012 to −0.178, *p* = 0.005) and paying more attention to neutral (β = −0.114, 95%CI: −0.195 to −0.032, *p* = 0.007) or negative (β = −0.317, 95%CI: −0.510 to −0.124, *p* = 0.005) information were negatively associated with an increased willingness to be a doctor after pandemic. The detailed information is shown in (Table 3).

**Table 3.** Predictors of changes in willingness to be a doctor in linear regression analysis

Independent variables	Unadjusted coefficient (95% CI)	Adjusted coefficient (95% CI)†
Covariates		
Age	−0.011(−0.022 to 0.000)*	−0.015(−0.025 to −0.004)**
The willingness to be a doctor before the COVID-19 pandemic	−0.084(−0.103 to −0.065)***	−0.107(−0.127 to −0.087)***
The severity of depressive symptoms	−0.007(−0.015 to 0.001)	−0.011(−0.019 to −0.003)**
The severity of anxiety symptoms	−0.010(−0.020 to −0.001)*	-
The severity of insomnia symptoms	−0.004(−0.012 to 0.004)	-
Factors		
Gender, ref. Men	−0.076(−0.162 to 0.009)	−0.099(−0.181 to −0.018)*
Ethnicity, ref. Chinese Han	−0.059(−0.164 to 0.046)	-
Education level, ref. Graduate students	0.086(−0.021 to 0.193)	-
Annual household income (¥)		
≤100 thousand	ref.	ref.
100–300 thousand	−0.126(−0.228 to −0.024)*	−0.080(−0.179 to 0.018)
>300 thousand	−0.311(−0.493 to −0.128)**	−0.261(−0.437 to −0.085)**
Habit of regular exercising, ref. No	0.002(−0.082 to 0.085)	-
Exposure to the most affected pandemic area, ref. No	0.005(−0.282 to 0.292)	-
Preferential attention toward pandemic information		
Negative	−0.271(−0.470 to −0.072)**	−0.317(−0.510 to −0.124)**
Neutral	−0.065(−0.149 to 0.019)	−0.114(−0.195 to −0.032)**
Positive	ref.	ref.
Receiving negative feedback from your families and friends who joined the frontline work, ref. No	−0.743(−1.176 to −0.310)**	−0.595(−1.012 to −0.178)**
Having relatives and friends with suspected or confirmed COVID-19, ref. No	−0.133(−0.355 to 0.089)	-
Following your own inclinations when choosing to major in medicine, ref. No	−0.081(−0.191 to 0.028)	-
Being interested in medicine, ref. No	−0.242(−0.384 to −0.101)**	-
Influenced by your elders (parents or other relatives) when selecting medicine as your major, ref. No	−0.054(−0.135 to 0.027)	-
Changes in professional satisfaction after the COVID-19 pandemic		
Less satisfied	−0.275(−0.432 to −0.119)***	−0.348(−0.501 to −0.195)***
No change	ref.	ref.
More satisfied	0.409(0.295 to 0.522)***	0.364(0.253 to 0.475)***

†Adjusted for age, sex, ethnicity, educational level, income level, habits of exercise, geographic location, aspects of information attention, negative feedback exposure, infection status of COVID-19 among friends and relatives, the motivation in selecting medicine, changes of professional satisfaction, symptoms of depression, anxiety, and insomnia. *p < 0.05, **p < 0.01, ***p < 0.001

## Factors independently associated with increased or decreased willingness to major in respiratory medicine and infectious diseases

As shown in (Table 4), individuals who had relatives with suspected or confirmed infection (OR = 3.368, 95%CI: 1.751 to 6.478, *p* < 0.001) and higher level of anxiety symptoms (OR = 1.099, 95%CI: 1.061 to 1.139, *p* < 0.001) were more likely to decrease willingness to select respiratory medicine and infectious diseases as their future career specialties. Individuals who chose medicine according to their own wishes (OR = 0.570, 95%CI: 0.387 to 0.840, *p* = 0.004) were less likely to decrease willingness to select respiratory medicine and infectious diseases as their future majors after the pandemic. Compared with paying more attention to positive  pandemic information, paying more attention to negative (OR = 2.301, 95%CI: 1.219 to 4.343, *p* = 0.010) COVID-19 information was associated with decreased willingness to specialize in respiratory medicine and infectious diseases. Those had decreased (OR = 2.242, 95%CI: 1.325 to 3.795, *p* = 0.003) willingness to be a doctor after the COVID-19 pandemic were also more likely to decrease willingness to major in respiratory medicine and infectious diseases.

**Table 4.** Predictors of changes in willingness to specialize in respiratory medicine and infectious diseases in binary logistic regression analysis

Independent variables	Decreased willingness		Increased willingness	
	Unadjusted OR (95% CI)	Adjusted OR (95% CI)†	Unadjusted OR (95% CI)	Adjusted OR (95% CI)†
Covariates				
Age	1.078(1.037 to 1.120)***	-	0.948(0.908 to 0.989)*	-
The severity of depressive symptoms	1.095(1.065 to 1.125)***	-	0.997(0.962 to 1.033)	-
The severity of anxiety symptoms	1.138(1.102 to 1.176)***	1.099(1.061 to 1.139)***	0.997(0.968 to 1.027)	-
The severity of insomnia symptoms	1.095(1.065 to 1.125)***	-	0.994(0.965 to 1.024)	-
Factors				
Gender, ref. Man	0.905(0.649 to 1.262)	-	0.620(0.463 to 0.831)*	0.640(0.471 to 0.869)**
Ethnicity, ref. Chinese Han	1.794(1.094 to 2.942)*	1.592(0.937 to 2.705)	0.745(0.527 to 1.053)	-
Education level, ref. Graduate students	0.550(0.384 to 0.788)**	-	2.442(1.461 to 4.082)***	2.180(1.290 to 3.683)**
Annual household income (¥)				
≤100 thousand	ref.	-	ref.	-
100–300 thousand	1.351(0.925 to 1.974)	-	0.626(0.414 to 0.947)*	-
>300 thousand	2.321(1.321 to 4.081)**	-	0.805(0.395 to 1.642)	-
Habit of regular exercising, ref. No	0.786(0.560 to 1.105)	-	1.812(1.359 to 2.417)***	1.559(1.155 to 2.105)**
Exposure to the most affected pandemic area, ref. No	1.448(0.553 to 3.790)	-	0.692(0.209 to 2.293)	-
Preferential attention toward pandemic information				
Negative	3.626(2.054 to 6.404)***	2.301(1.219 to 4.343)*	0.177(0.043 to 0.734)*	0.191(0.045 to 0.803)*
Neutral	1.701(1.219 to 2.374)**	1.405(0.983 to 2.007)	0.540(0.391 to 0.746)***	0.575(0.412 to 0.803)**
Positive	ref.	ref.	ref.	ref.
Receiving negative feedback from your families and friends who joined the frontline work, ref. No	5.136(1.843 to 14.309)**	-	-	-
Having relatives and friends with suspected or confirmed COVID-19, ref. No	3.912(2.162 to 7.078)***	3.368(1.751 to 6.478)***	2.067(1.250 to 3.417)**	-
Following your own inclinations when choosing to major in medicine, ref. No	0.455(0.319 to 0.650)***	0.570(0.387 to 0.840)**	1.598(1.126 to 2.267)**	-
Being interested in medicine, ref. No	0.546(0.349 to 0.854)**	-	5.291(1.936 to 14.459)**	5.795(2.087 to 16.089)***
Influenced by your elders (parents or other relatives) when selecting medicine as your major, ref. No	1.338(0.977 to 1.834)	-	1.612(1.209 to 2.149)**	1.543(1.145 to 2.080)**
Changes in willingness to be a doctor after the COVID-19 pandemic				
Decreased willingness	6.224(4.065 to 9.530)***	4.370(2.747 to 6.953)***	1.334(0.723 to 2.461)	1.387(0.720 to 2.671)
No change	ref.	ref.	ref.	ref.
Increased willingness	1.081(0.615 to 1.900)	0.769(0.419 to 1.411)	1.646(1.092 to 2.482)*	1.738(1.110 to 2.720)*
Changes in professional satisfaction after the COVID-19 pandemic				
Less satisfied	3.528(2.218 to 5.609)***	2.242(1.325 to 3.795)**	1.616(0.933 to 2.798)	1.464(0.817 to 2.623)
No change	ref.	ref.	-	ref.
More satisfied	1.425(0.913 to 2.225)	1.403(0.867 to 2.270)	1.938(1.350 to 2.783)***	1.870(1.271 to 2.750)**

†Adjusted for age, sex, ethnicity, educational level, income level, habits of exercise, geographic location, aspects of information attention, negative feedback exposure, infection status of COVID-19 among friends and relatives, the motivation in selecting medicine, changes of professional satisfaction and the willingness to be a doctor, symptoms of depression, anxiety, and insomnia. *p < 0.05, **p < 0.01, ***p < 0.001

However, individuals who were female (OR = 0.640, 95%CI: 0.471 to 0.869, *p* = 0.004) and pay attention to neutral (OR = 0.575, 95%CI: 0.412 to 0.803, *p* = 0.001) or negative (OR = 0.191, 95%CI: 0.045 to 0.803, *p* = 0.024) information are less likely to increase willingness to select respiratory medicine and infectious diseases as their future majors after the pandemic. Students who had a habit of regular exercising (OR = 1.559, 95%CI: 1.155 to 2.105, *p* = 0.004), were undergraduate students (OR = 2.180, 95%CI: 1.290 to 3.683, *p* = 0.004), were interested in medicine (OR = 5.795, 95%CI: 2.087 to 16.089, *p* = 0.001), and were influenced by elders when selecting medicine as major (OR = 1.543, 95%CI: 1.145 to 2.080, *p* = 0.004) were more likely to select respiratory medicine and infectious diseases as their future majors after the pandemic. Those who became more satisfied with their majors after the COVID-19 pandemic (OR = 1.870, 95%CI: 1.271 to 2.750, *p* = 0.001), and had increased (OR = 1.738, 95%CI: 1.110 to 2.720, *p* = 0.016) willingness to be a doctor after the COVID-19 pandemic were also more likely to choose respiratory medicine and infectious diseases as their future specialties.

## Discussion

The present study investigated the inclinations of Chinese medical students to be clinical doctors in the future and any changes in their willingness to specialize in respiratory medicine and infectious diseases after the COVID-19 pandemic. Approximately one-fifth of the participants reported that the COVID-19 pandemic affected their inclination to be a clinical doctor or to choose respiratory medicine and infectious diseases as their future specialties, with relatively more students showing increased willingness and determination to become a clinical doctor and to choose respiratory medicine and infectious diseases. Several COVID-19-related factors associated with career and specialty choices were identified, including major satisfaction and interests, levels of depression and anxiety, and types of pandemic information exposed. These findings revealed that COVID-19 could affect the career choice and major selection of medical students in both positive and negative ways, suggesting that developing career education and guidance, making appropriate training policies and timely monitoring of the psychological status of medical students during and after the pandemic are of great importance to expand human resources for the healthcare sector in China.

The COVID-19 pandemic posed significant obstacles to the training progress of medical students. Closures of universities have substantially prevented medical students from obtaining theoretical knowledge and participating in clinical clerkships, which may affect their career and specialty choices[12]. Although an internet-based platform was established to address issues related to the cancellations of face-to-face teaching in medical schools[25], the suspension of clinical clerkships and rotations for senior medical students would be difficult to replace using the internet. For graduate students, closures of universities and laboratories have substantially hindered students’ ability to complete graduation projects and experiments, which may exert additional pressure on these students, leading to reduced commitment to medical careers and decreased academic performance[26].

Depression and anxiety have become pervasive among medical students during the COVID-19 pandemic [10,27]. The present study found that the prevalence rates of symptoms of anxiety, depression and insomnia in students with decreased willingness to be a doctor were 59.5%, 56.3% and 38.1%, respectively, and were higher than in other reports conducted before the COVID-19 pandemic[27]. These findings were consistent with previous studies that found that major public health outbreaks could further increase the degree of anxiety and stress in medical students [7,28]. Interestingly, we found that the severity of depression symptoms was negatively associated with willingness to be a doctor, and the severity of anxiety symptoms was associated with decreased willingness to select majors in respiratory medicine or infectious disease after the COVID-19 pandemic. This may occur because psychological problems could enhance burnout, affect medical career interest and increase career choice regret and dropout rates among medical students [10,11]. These findings highlight the importance of improving the mental health of medical students and hopefully provide better control and prevention of pandemic infection. On the other hand, there was also a seemingly positive influence of the pandemic on the determination and enhanced interest in medical study in a group of students. Such a positive influence was also witnessed during the previous SARS-CoV epidemic, in which the general population reported enhanced positive mental health impacts[29].

The present study found that those who obtained high satisfaction with their profession were associated with increased enthusiasm for studying medicine after the pandemic. Major satisfaction refers to an individual’s evaluation of their current majors and major-related future careers, which is critical in the career development process [30,31]. Besides, we also found that being interested in medicine was associated with increased willingness while making choice of medical career according to their own wishes was less likely to decrease willingness to major in respiratory medicine and infectious diseases. Academic interest is associated with career choice motivation[32]. ‘Match with interest’ is proved to be more important than job characteristics, major attributes, and other psychological and social factors when choosing a major[33]. In addition, interests are found to be the primary factor in choosing infectious disease specialties among graduating medical residents and are associated with medical students’ career choice motivation [3,32]. Therefore, students should consider their interests when choosing medicine majors, and medical schools should offer diversified and individualized courses in clinical medicine and add career planning sessions to create an appropriate learning environment for medical students, enhance their interest in medicine, increase their major satisfaction and professional belief, and help them make their career specialty decisions.

In the present study, we found that exposure to COVID-19-related information could affect medical students’ career and major selection. Paying more attention to positive information about the pandemic, e.g., the selfless dedication of healthcare workers and the respectable career choice of being a doctor, may reduce professional uncertainties. However, paying more attention to negative information about the COVID-19 pandemic was associated with a higher risk of psychological problems among both medical staff and students [16,34]. This study also found that individuals who had relatives with suspected or confirmed infection may be more likely to decrease willingness to choose respiratory medicine and infectious diseases as their majors. Given the negative effects of the COVID-19 pandemic on mental health and specialty choice, the education department should develop effective response measures for medical students, help them establish positive values and attitudes, and strengthen the role of professional ethics in education. In addition, an emerging combined subspecialty of critical care medicine and infectious diseases model may encourage more students to choose this field[35].

The present study has several limitations. First, as this study adopted a cross-sectional design, it remains unknown whether medical students’ attitudes towards career choice will change when the pandemic is under control or after graduation. Second, we did not use standardized, interviewer-rated, and formally validated questionnaires when assessing career and specialty choices. The factors that may influence the career choice of medical students have not been sufficiently investigated. Thus, qualitative studies with a comprehensive understanding of the potential reasons for changes in career choices are needed in the future. Third, the sample size in the current study was modest, and this convenience sample was unlikely to be representative of the group of medical students, which may bias the results. Fourth, although the sample characteristic of respondents was similar with previous online cross-sectional study[36], we cannot obtain the sample characteristic of non-respondents in this online cross-sectional survey, no comparison between respondents and non-respondents can be conducted, there maybe has non-response bias. Fifth, only changes in choosing a career as a doctor and as a respiratory or infectious disease physician before and after the pandemic were investigated, and other majors were not included.

## Conclusions

The present study found that the COVID-19 pandemic and its related factors, such as exposure to COVID-19-related information, anxiety and depression levels, and changes in professional satisfaction, affect choices to enter a medical career and specialize in respiratory medicine and infectious diseases among medical students. Medical educational institutions should pay more attention to the psychological and occupational impacts of COVID-19 on medical students.
